# The effects of face coverings, own-ethnicity biases, and attitudes on emotion recognition

**DOI:** 10.1186/s41235-022-00400-x

**Published:** 2022-07-02

**Authors:** Holly Cooper, Amrit Brar, Hazel Beyaztas, Ben J. Jennings, Rachel J. Bennetts

**Affiliations:** Division of Psychology, College of Health, Medicine, and Life Sciences, Brunel University London, Kingston Lane, Uxbridge, UB8 3PH UK

**Keywords:** Face coverings, Own-ethnicity bias, Mask attitudes, Emotion recognition

## Abstract

As a result of the COVID-19 pandemic, face coverings were introduced as a safety measure in certain environments in England and some research suggests that they can affect emotion recognition. Factors such as own-ethnicity bias (e.g. whether people perceiving and expressing emotions are of the same ethnicity) and social biases are also known to influence emotion recognition. However, it is unclear whether these factors interact with face coverings to affect emotion recognition. Therefore, this study examined the effects of face coverings, own-ethnicity biases, and attitudes on emotion recognition accuracy. In this study, 131 participants viewed masked and unmasked emotional faces varying in ethnicity and completed a questionnaire on their attitudes towards face masks. We found that emotion recognition was associated with masks and attitudes: accuracy was lower in masked than unmasked conditions and attitudes towards masks Inside and Outside were associated with emotion recognition. However, a match between perceiver and stimulus ethnicity did not have a significant effect on emotion recognition. Ultimately, our results suggest that masks, and negative attitudes towards them, were associated with poorer emotion recognition. Future research should explore different mask-wearing behaviours and possible in-group/out-group biases and their interaction with other social cues (e.g. in-group biases).

## Significance statement

The current study found a significant effect of masks and attitudes towards masks on emotion recognition accuracy. We found masks were negatively associated with emotion recognition accuracy for all emotional expressions. There was an increase in confusion of emotions with other emotional states when masked, the biggest misclassification being fear with surprise. We found that a match or mismatch of stimuli and participant ethnicity did not affect emotion recognition accuracy: mask effects were not heightened when people were recognising emotion for other ethnicity stimuli faces compared to their own ethnicity. The attitude findings raise the possibility that mask attitudes could be acting as a social grouping cue: if you have negative attitudes towards mask wearing, you may see those who wear masks as different to you; therefore, they become your social “out-group”. This could result in these individuals processing masked faces differently than unmasked faces, which in turn could negatively impact emotion recognition. We found that negative attitudes to people who wear masks outside (where it was not mandatory) correlated with worse recognition accuracy in masked faces. Previous research found ethnicity acts as a social grouping cue, but in this case masks may have acted as one. Although the findings do suggest that the effects of masks were largely perceptual, this proposes policy recommendations of using modified masks which could spare the emotion recognition deficits caused by masks.

## Introduction

In response to the COVID-19 pandemic, the government made face coverings mandatory in England in indoor settings such as shops, transport, and restaurants (Department of Health & Social Care, 2021). By August 2020, over one hundred other countries also introduced mask mandates (Felter & Bussemaker, 2020). Face coverings were found to be useful in preventing the spread of the virus (Asadi et al., 2020). However, face masks can make it difficult to extract information from a face, so we need to know how this affects emotion recognition. Emotion recognition is vital in establishing and maintaining relationships (Grossmann, 2017) as well as ensuring we act appropriately in certain situations (Grundmann et al., 2021). There is increasing evidence that masks impact face recognition (Noyes et al., 2021) and social judgements (Biermann et al., 2021). Examining these effects is crucial to understanding if and how face masks can impact our social interactions.

One important aspect of social interactions is emotion recognition. Van Kleef (2009) proposed the emotion-as-social-information model. This model states that emotion recognition is vital as emotional expressions can prompt cognitive processes which then lead to actions. For example, passengers on a bus looking angrily at you could prompt you giving up your seat for an elderly passenger. As each emotion conveys different information, the accuracy of emotion recognition is crucial to act appropriately in situations (Grundmann et al., 2021). Another relevant theory is the evolutionary perspective (Al-Shawaf et al., 2016). We have adapted to recognise anger, disgust, and fear quickly and accurately. This helps us avoid harmful situations, such as ingesting something toxic or fleeing from danger, for our survival (Al-Shawaf et al., 2016). Therefore, this suggests the recognition of these emotions should be fast and accurate.

There are good reasons to predict that emotion recognition would be affected by masks. Faces carry important cues to help us recognise emotional expressions, for some emotions these cues are carried in the lower region of the face. This has been explored using various methods. Point light displays (faces covered in black make up and white spots) (Bassilli, 1979), the tile method (48 tiles sequentially uncovered and participants stop the sequence when they recognise the emotion) (Wegrzyn et al., 2017), and eye tracking (Eisenbarth & Alpers, 2011) all agree that sadness, fear, and anger relied on the eyes for recognition and disgust and happiness relied on the mouth. These papers offer converging evidence that certain facial features are vital in specific emotion recognition. Given that face coverings cover the lower region of the face, the effects on emotion recognition are likely to vary across emotion. Specifically, the emotions relying on the lower region of the face (disgust and happiness) may be most affected by face coverings. Also, for face recognition, configural processing (i.e. extracting information about the spatial relationships between features, as well as the features themselves) plays an important role (Pascalis et al., 2011). Previous research also found that configural processing facilitates emotion recognition too (Bombari et al., 2013; Durand et al., 2007). Therefore, face coverings, which conceal the nose and mouth, would affect the information needed to process the spatial relations between the eyes and other features and possibly affect recognition. In support of this, Freud et al. (2020) found that face masks changed the way in which faces were perceived. Masked faces resulted in a weaker inversion effect, which may reflect reduced configural processing (Rossion, 2008).

While it is likely that specific emotions will be affected by face masks, other areas of the face, unaffected by masks, still contribute substantially to emotion recognition. Previous papers found the eyes were important for accurate emotion recognition. The widely used Reading the Mind in the Eyes Test (Baron-Cohen et al., 2001) was used to explore emotion recognition from the eye region. Participants see 36 photographs of the eye region and decide which emotion is being expressed (Seo et al., 2020). Schmidtmann et al. (2020) used the Reading the Mind in the Eyes Test and presented the stimuli across eight presentation times (12.5–100 ms). They found that participants could recognise subtle differences between facial expressions. This supports the idea that the eye region plays a key role in social interactions and provides a rich source of information about our emotional states. In support of this, a review concluded that the recognition of emotional states is collated from the eye region and provides a vital basis for initiating, maintaining, and regulating social interactions with others (Grossmann, 2017). This suggests that the area of the face that remains uncovered by face masks (upper face region) is sufficient to support some level of emotion recognition accuracy. Therefore, although masks may lead to a decrease in emotion recognition accuracy, it is unlikely that emotion recognition will be completely abolished by the use of face coverings.

Masks may also affect emotion recognition due to the increased confusion of specific emotions with others when faces are partially covered (e.g. Carbon, 2020; Fischer et al., 2012). These studies reinforce the importance of examining the effects of masks on emotion recognition separately for individual emotions. In sum, a thorough understanding of the effects of masks on emotion recognition requires us to look at both overall accuracy and potential confusions/misidentifications across a broad range of emotions, the approach adopted in the current study.

### Masks and emotion recognition

Since the pandemic, there has been a surge in research into face coverings and emotion recognition. Noyes et al. (2021) examined emotion recognition performance with occlusions (either face coverings or sunglasses) or no occlusions, in both typical recognisers and super-recognisers (those highly skilled at face recognition; Russell et al., 2009). They found that any occlusion of the face resulted in reduced accuracy for both face identification and emotion recognition. Errors were highest in the mask condition, showing the negative impact masks have on emotion recognition abilities. However, unlike many emotion recognition studies, Noyes et al. (2021) used real images of people wearing masks and sunglasses rather than editing images. Whilst using real stimuli is more naturalistic, it means that the control (non-occluded) images are not perfectly matched to the occluded ones.

In support of mask effects, Grundmann et al. (2021) found that overall emotion recognition accuracy declined from 69.9% for unmasked to 48.9% for masked faces. Carbon (2020) investigated this and showed 41 participants’ six emotional expressions: angry, disgusted, fearful, happy, sad, and neutral. These were either fully visible or partially covered by a mask. All emotions, except fear, were repeatedly confused with neutral, and sadness and anger were confused with disgust as well as neutral. Notably, Carbon (2020) found that the greatest emotion misidentification was disgust with anger in the masked condition (occurring in nearly 38% of the masked stimuli compared to only 2% of the unmasked stimuli). This study showed that masks increase confusion when faces were partially visible. A similar study (Bani et al., 2021) reported comparable results of emotion recognition for masked faces (with the exception of fear) being reduced in both low- and high-intensity emotions. The finding of fear being unaffected in these studies is in line with the evolutionary perspective, stating that the greater sensitivity is for our survival (Kostić & Chadee, 2015). Fear being unaffected by masks could have occurred because of the distinct perceptual characteristics of the stimuli: fear is expressed with wide eyes and most other emotions are not. However, an issue with these studies is the exclusion of surprise. This is because there may have been some confusion between surprise and fear, which share similar perceptual characteristics.

In contrast, a study using a younger sample concluded masks may only have a minimal impact on social interactions. Ruba and Pollak (2020) recruited a racially diverse sample of children aged 7 to 13 years old. The stimuli were either not covered, wearing sunglasses, or surgical masks. They found the children’s emotion inferences about masked faces compared to unmasked faces were still above chance. This suggests the children’s expression recognition was minimally affected by masks. A limitation of the study is the editing of the stimuli. The masks covered the nose and mouth but did not reach the sides of the face. This is not representative of real-life mask wearing. However, the different results across various ages highlight the need to explore mask effects across a broader sample and take different individual differences into account.

While most research has focused on establishing the presence and size of mask effects on emotion recognition, it is important to determine the locus of those effects. In other words, are the effects of masks on emotion recognition purely perceptual (the result of a lack of relevant visual information), or could they also be driven by social biases? Some preliminary findings suggest that the effects of masks are at least partially perceptual. Marini et al. (2021) used standard medical face masks, transparent face masks which showed the mouth area, and no masks. They found that transparent masks spared the accuracy of emotion recognition, creating minimal to no effect, compared to medical masks. This indicates at least some effects of masks are perceptually based. However, it is also possible that the presence of a face covering could induce social biases in the observer towards the mask wearer—a factor that was not considered in Marini et al.’s (2021) research.

There is recent evidence demonstrating that masks can create in- and out-groups. During the Prisoner’s dilemma (a cooperation task), both mask and non-mask wearers showed an in-group bias when information about mask usage was known (Powdthavee et al., 2021). Mask wearers were more cooperative to other mask wearers, and non-mask wearers were more cooperative to other non-mask wearers. This supports the idea that masks can act as a social grouping cue. There is also evidence that other facial occlusions—specifically, religious face coverings—can influence emotion perception for partially covered faces. Kret and De Gelder (2012) presented participants with faces that were obscured by a religious face covering (a niqab) or other face coverings (cap and scarf). Even though the niqab condition and the cap and scarf condition covered the same regions of the face, the faces in the niqab condition were attributed negative emotions more frequently than in the cap and scarf condition. Similarly, Fischer et al. (2012) explored the perception of emotions using female stimuli whose faces were either fully visible, covered by a niqab, or partially covered (showing only the eye region). They found that in the niqab condition happiness was recognised less, and confused more with negative emotions, compared to other conditions. Together, these studies suggest that the effects of face coverings can be more complex than simple perceptual occlusion, and it is important to examine how different factors (e.g. characteristics of the perceiver or expresser) might interact with face masks to affect emotion recognition. Therefore, the aim of the current research is to investigate if individual differences, specifically social group cues (ethnicity) and attitudes towards masks, modulate the effects of masks.

### In-group/out-group biases and emotion recognition

Previous research provides evidence for own ethnicity, own culture, and in-group identity being an important factor in emotion recognition. Elfenbein and Ambady (2002) performed a meta-analysis examining own-group biases in emotion recognition. They concluded that accuracy was higher when emotions were expressed and recognised by members of the same national, ethnic, or religious group. This suggests an in-group advantage. However, the advantage was smaller for cultural groups with greater exposure to other groups. More recent research supports the idea that in-group ethnicity effects can be attenuated by exposure. Yan et al. (2016) asked Chinese and White participants to sort Chinese and White faces by identity and expression. They found evidence of an in-group bias as participants were better at sorting their own ethnicity. However, there was also a considerable amount of cross-cultural agreement. Another study that found cross-cultural agreement was Soto and Levenson (2009). They tested the emotion recognition accuracy of 161 college students. They found no significant difference in performance when viewing in-groups compared to out-groups. A possible explanation for why both papers found cross-cultural agreement could be because they used student samples. Students tend to have more exposure to diverse populations, and therefore it is rarer to find a difference in performance between in-groups and out-groups.

In-group and out-group biases affect accuracy of emotion recognition, but they can also bias perceptions in more specific ways. For example, research using religious face coverings suggests that European participants attribute more negative emotions to faces covered by religious coverings, compared to those covered by a cap and scarf (Kret & De Gelder, 2012). Research using religious face coverings agrees that group biases can negatively impact emotion recognition beyond the simple perceptual effects of a face being covered (Fischer et al., 2012). It is possible that similar interactions could occur with face masks. Ethnicity is a social grouping cue, and masks could also be a social grouping cue. This means that emotion recognition accuracy for masked faces may also be affected by perceived biases. For example, people who wear masks could prefer others who wear masks and therefore this would create an in-group advantage for masked conditions. This is similar to how ethnicity biases affect emotion recognition and relates the two factors in this instance. Consequently, research on face coverings should include both masked and unmasked faces varying in ethnicities to see if face masks increase, decrease, or have no effect on the out-group disadvantage for emotion recognition.

To date, research on mask effects has not taken own-ethnicity biases into account. Therefore, it is difficult to predict whether the effects of masks would interact with any in-group biases, such as ethnicity. However, in-group advantages for emotion recognition have often been linked to social–motivational processes (Young & Hugenberg, 2010). In short, it is claimed that individuals allocate more attention to in-group faces, whereas they disengage from processing out-group faces. Young and Hugenberg claim that the additional attention to in-group faces occurs after initial, automatic emotion processing, thereby improving expression recognition above “baseline” levels. In the context of masks, this could mean that individuals attend to in-group faces (in this case, faces of the same ethnicity as theirs) more than out-group faces, thus attenuating the effects of masks.

### Attitudes and emotion recognition

Attitudes have been associated with emotion recognition in general. There is some evidence that an individual’s attitude towards a person or group is associated with their attribution of emotions to members of that group. Much of the previous research has involved ethnicity. For example, some studies have reported that White participants, who according to an implicit association test, were high in implicit prejudice, reported a higher intensity rating of an angry face if it was categorised as Black (Hutchings & Haddock, 2008). This suggests that their negative attitudes were associated with less accurate emotion perception. Consistent with these findings, Wang et al. (2014) also found implicit biases were associated with perceived intensity of emotions. The implicit attitudes of Chinese participants, towards White people, biased their perception and judgement of emotional intensity. Pro-Chinese and anti-White implicit attitudes were associated with higher intensity ratings of negative expressions (anger, fear, and sadness) in White faces. In further support, Van Hiel et al. (2019) explored emotional abilities and right-wing and prejudiced attitudes. They asked participants to complete an emotional abilities and right-wing and prejudiced attitudes questionnaire. They found that emotional abilities were negatively related to right-wing and prejudiced attitudes. This suggests that attitudes are associated with emotional abilities. These findings together provide evidence for attitudes being associated with poorer emotional abilities when viewing out-group stimuli. Currently, though, it is unclear whether similar effects may be observed for other attitudes and biases, such as attitudes towards masks.

Attitudes towards masks in the UK vary considerably. Some people accept face masks and the safety that comes with them, while others deem them “oppressive”, as voiced in the anti-mask riots (Taylor & Asmundson, 2021). These attitudes have been shown to affect behaviour and health outcomes. For example, attitudes towards mask wearing were associated with face mask purchase intentions in Pakistan (Shah et al., 2020), conformity to masculine norms (Mahalik et al., 2021), as well as reductions in COVID-19 cases (Adojdah et al., 2021). Furthermore, in a recent study conducted in Germany, attitudes towards masks correlated with trust judgements: individuals with more negative attitudes tended to rate masked faces as less trustworthy than unmasked faces (Biermann et al., 2021). This research shows that attitudes towards masks can have a significant effect on other variables. Currently, though, we do not know whether mask attitudes could increase or decrease the effects of masks on emotion recognition.

### Summary and research aims

The purpose of this research is to explore how face coverings affect emotion recognition performance. We are particularly interested in the effect of own-ethnicity biases and attitudes on masked and unmasked faces. Specifically, (1) whether mask effects are consistent or more evident when attempting to recognise emotions in individuals of a different ethnicity; and (2) if different attitudes towards masks and mask wearers affect masked emotion recognition.

To address these questions, we examined emotion recognition in masked and unmasked faces in three ways. Firstly, we analysed the overall effect of masks on emotion recognition accuracy and the patterns of confusions or misidentifications that occur across different emotions. In line with previous research (e.g. Carbon, 2020; Noyes et al., 2021), we hypothesise that masks will significantly reduce accuracy for emotion recognition compared to unmasked faces. Masks will also lead to different patterns of confusion/misidentification (e.g. higher confusion between the emotions and “neutral” faces). Secondly, we examined the effect of own-ethnicity biases by comparing recognition performance when participant and stimulus ethnicity was the same or different. Previous research suggests that a mismatch between perceiver and expresser ethnicity can influence emotion recognition (Elfenbein & Ambady, 2002). However, it is unclear whether these effects will be exacerbated by masks (e.g. will out-group biases increase more in masked conditions than unmasked). Finally, we examined the effect of attitudes towards masks on emotion recognition accuracy. Research on religious face coverings suggests that attitudes and biases can influence emotion recognition, over and above the effects of simple perceptual occlusion. Therefore, it is possible that individuals with strong negative or positive attitudes towards face masks may show different performance for masked faces. Collectively, these analyses replicate and strengthen the findings from previous research on masks and emotion recognition (e.g. Carbon (2020)). The analyses also extend on previous research by examining how individual differences in the mask wearer and perceiver can modulate the effects of masks on emotion recognition.

## Methods

### Participants

A total of 137 participants completed this study, but only 131 participants’ data were analysed. The data from six participants were excluded: two participants failed to respond within the time limit on more than 10% of trials (indicating a lack of engagement with the task), three participants did not consent to all sections of the consent form, and one participant’s data were incomplete. From the 131 participants (103 female; 27 male; 1 nonbinary, *M*_age_ = 20, *SD* = 2.01), 38 identified their ethnicity as White (29.0%), 21 identified as Black (16.0%), 37 identified as Asian/Pacific Islander (28.2%), and 35 selected “Other” (26.7%). Participants were recruited from the undergraduate psychology cohort at Brunel University London and took part in exchange for course credit. Informed consent was obtained from all participants before completing the study. Ethical approval was granted by the Research Ethics Committee at Brunel University London.

## Materials

### Emotional faces

**Fig. 1:**
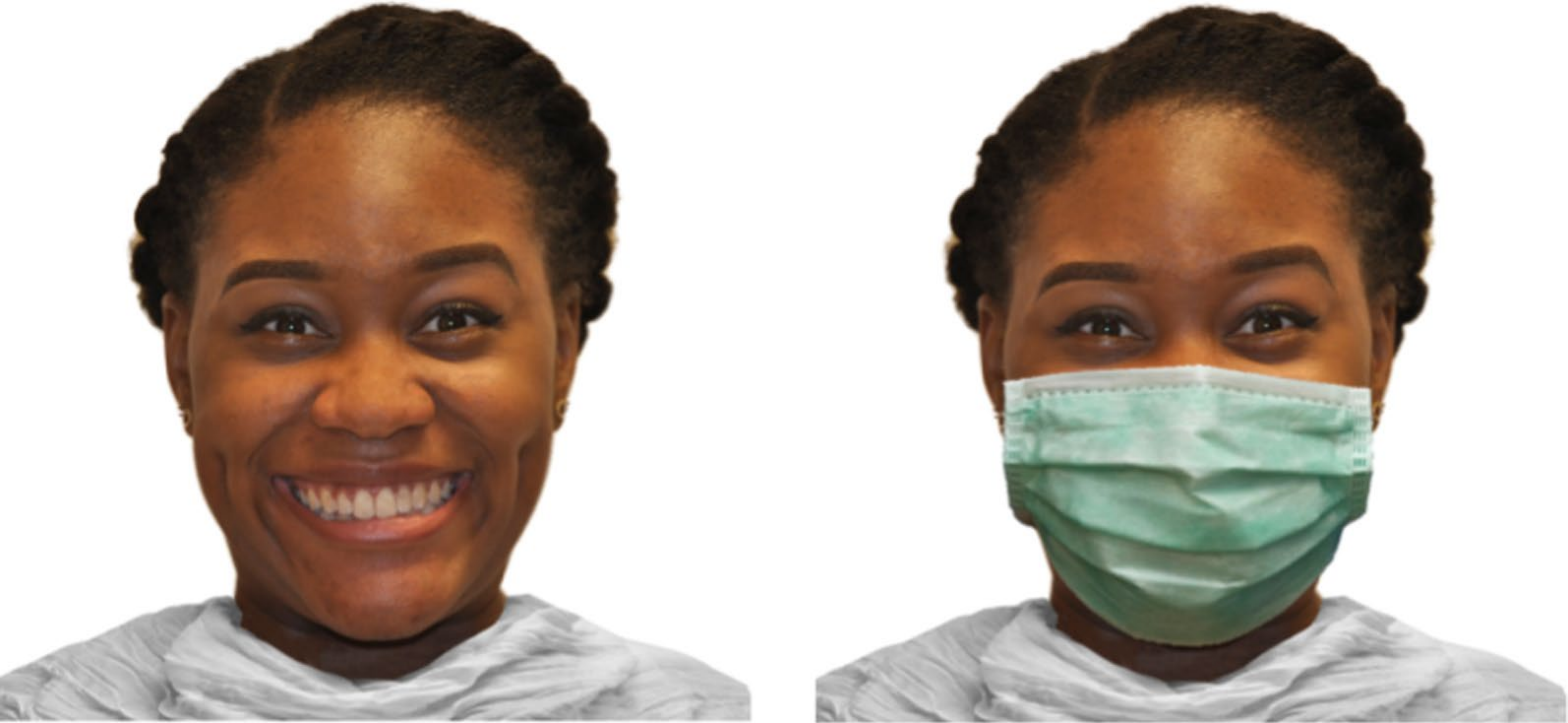
Examples of unmasked (left) and masked (right) stimuli both expressing the emotion of happiness

The stimuli were taken from RADIATE, a validated database (Conley et al., 2018). This database was chosen because it is a large, ethnically diverse set of emotional faces with good reliability and validity. Colney et al. (2018) established there was consistency between the first and second ratings of the emotional expressions with a mean reliability of 0.70 (*SD* = 0.16). Also, the Kappa scores (overall measure of agreement between labels and actors intended emotional expression) were substantial (*M* = 0.65, *SD* = 0.19). This suggests the stimuli accurately expressed their intended expression (Conley et al., 2018). The stimuli chosen from this database were selected as a result of their average accuracy ratings. Each average was above 70% accuracy (see Appendix 1 for examples of the stimuli used in the current experiment and Appendix 2 for the mean accuracy of identification as per Conley et al. (2018)). A total of twenty-four identities were chosen and included three ethnicities (Asian, Black, and White) with 4 females and 4 males chosen for each ethnicity. For each ethnicity, seven images were selected (one each displaying happy, sad, angry, fear, disgust, surprise, and neutral expressions). The original unmasked stimuli were duplicated to edit face masks onto them using the website Photopea (examples are in Fig. 1). Four different face masks were selected from online sources (Google image searches); all had the same basic shape and covered the same areas of the face but they differed in colour and style. This was to make the task somewhat more realistic. The different masks were distributed equally across the stimuli (but each individual was always depicted with the same mask). In total, there were 336 emotional faces: four identities x three ethnicities x two gender x seven emotions x two images (masked/unmasked).

### Attitudes towards masks

At the end of the experiment, participants were presented with a 13-item questionnaire. This explored their mask-wearing behaviour and attitudes towards mask wearing (see Appendix 3 for full questionnaire). In the first section, participants were asked to rate how likely they were to wear masks in different environments on a 7-point Likert scale (1 = Never; 4 = About half the time; 7 = Always). In the second and third sections, participants were asked how they would rate their attitude towards someone who was wearing a mask (Sect. 2) and was NOT wearing a mask (Sect. 3) on a 7-point Likert scale (1 = Extremely negative; 4 = Neutral; 7 = Extremely positive). In each section, ratings were collected for four different environments: (a) on public transport; (b) in shops/businesses (when not eating); (c) in other enclosed/inside environments with multiple people inside (e.g. lecture halls); and (d) outside (e.g. walking down the street). Participants were also asked to provide their most common reason for not wearing a face mask from a drop-down list. This included reasons such as “I find it hard to breathe”, “I find it hard to communicate”, “I have an exemption”, “I am with someone who has an exemption”, “I forget to bring/wear a face covering”, “I just don’t want to”, and “I always wear a face covering”.

### Design

The independent variables were stimuli ethnicity (Asian, Black, White), face coverings (masked, unmasked), the emotion expressed (happy, sad, angry, fear, disgust, surprise, and neutral), attitudes towards masks, and participant ethnicity. The dependent variable was emotion recognition accuracy. The study employed a within-subjects design.

### Procedure

Participants were given instructions explaining their task was to identify the emotional expression displayed on each face. It would be displayed for one second, and they would have six seconds to select an answer. They were instructed to try and answer as quickly and accurately as possible, and told that if they waited too long the experiment would skip onto the next face. Before the main task, participants completed seven practice trials (which used faces not displayed in the main experiment). Order of presentation of the practice trials was randomised. They then moved onto the main task which consisted of a face expressing an emotion, presented in the middle of the screen. Order of presentation of trials in the main task was also randomised. The options for the answers were displayed underneath and were the basic six emotions and neutral. Participants responded by clicking the button corresponding to what they believed was the correct answer. Participants viewed half the faces with masks and half the faces without masks (the masked/unmasked faces were counterbalanced between participants). In total, participants completed 168 trials (7 × emotions; 3 × ethnicities; 2 × mask conditions; 2 × genders; 2 × identities).

Following the emotion task, participants completed the mask attitudes questionnaire.

The materials are available in this link (https://osf.io/57nfe/), and a fully programmed version of the experiment is available upon request from the authors.

## Results

The results are divided into three subsections. The first section examines the overall impact of masks, and the second and third examine the impact of own-ethnicity effects (2) and mask-wearing attitudes (3) on masked and unmasked emotion recognition.

### The effects of masks on emotion recognition

Descriptive statistics are presented in Table 1; the values represent mean performance (proportion correct) over all participants (*N* = 131) per emotion condition.

**Table 1:** Mean and SD emotion accuracy (proportion correct) for each emotion, masked and unmasked

	Masked		Unmasked		Average	
	Mean	SD	Mean	SD	Mean	SD
Angry	0.68	0.23	0.75	0.22	0.72	0.21
Disgust	0.21	0.14	0.77	0.21	0.49	0.14
Fear	0.29	0.19	0.46	0.22	0.37	0.18
Happy	0.65	0.19	0.93	0.14	0.79	0.15
Neutral	0.87	0.17	0.84	0.18	0.86	0.16
Sad	0.31	0.18	0.74	0.18	0.53	0.15
Surprise	0.67	0.21	0.79	0.20	0.73	0.17

A linear mixed effects model was employed to explore the effect of masks on emotion recognition accuracy. The fixed factors were mask condition and emotions, and the random variables were participant and actor (i.e. the identity of the individual expressing the emotions). The fixed factors had significant effects of mask condition, *F* (25.9) = 245.0, *p* < 0.001, and emotion, *F* (30.0) = 80.2, *p* < 0.001. There was also a significant interaction between masks and emotion, *F* (21,095.9) = 250.8, *p* < 0.001. Fixed effects parameter estimates are presented in Table 2. A complete table with fixed effects and random components is found in Appendix 4.

**Table 2:** Fixed effects table for the mask and emotion linear mixed effects model

Fixed effects	Estimate	Standard error	95% CI	*t*	*p*
Mask condition (unmasked–masked)	0.23	0.02	0.21, 0.26	15.7	< 0.001
Emotion 1 (anger–neutral)	− 0.14	0.04	− 0.21, − 0.06	− 3.7	0.001
Emotion 2 (disgust–neutral)	− 0.37	0.04	− 0.44, − 0.30	− 10.5	< 0.001
Emotion 3 (fear–neutral)	− 0.49	0.04	− 0.57, − 0.41	− 12.2	< 0.001
Emotion 4 (happy–neutral)	− 0.07	0.04	− 0.15, 0.02	− 1.5	0.139
Emotion 5 (sad–neutral)	− 0.33	0.04	− 0.41, − 0.26	− 8.8	< 0.001
Emotion 6 (surprise–neutral)	− 0.13	0.03	− 0.19, − 0.06	− 3.8	< 0.001
Mask condition * emotion 1	0.12	0.02	0.08, 0.16	6.3	< 0.001
Mask condition * emotion 2	0.60	0.02	0.57, 0.64	31.9	< 0.001
Mask condition * emotion 3	0.22	0.02	0.18, 0.25	11.3	< 0.001
Mask condition * emotion 4	0.32	0.02	0.28, 0.36	16.9	< 0.001
Mask condition * emotion 5	0.48	0.02	0.44, 0.51	25.4	< 0.001
Mask condition * emotion 6	0.15	0.02	0.11, 0.19	7.9	< 0.001

The variance of participant was 0.01 (ICC = 0.072) and the variance of actor was 0.01 (ICC = 0.032), which are small. Actor variance in the mask condition (< 0.01) and across emotions (ranging from 0.02 to 0.04) and participant variance in the mask condition (< 0.01) across emotions (ranging from 0.01 to 0.03) were also small. A comparison of the emotions found significant differences between all emotions, except anger and happy, anger and surprise, disgust and sad, happy and surprise, neutral and happy (all *p* values were 1.000).

As a result of the variance in the random effects of masks across actors and participant both being below 1%, it suggests the effect of actor and participant on the effect on masks was slight. The overall effect of masks was significant (*p* < 0.001), suggesting that the effects of masks were still significant even when variability between actors and participant was included in the model. There was a minimal effect of actor and participant on the overall linear mixed model, and there was a limitation of the nested design (i.e. as each participant only viewed the stimuli from each actor in one condition—masked or unmasked—the difference between masked and unmasked could not be separated out by actor). As such, subsequent analyses examining the mask effect were carried out as ANOVAs.

**Fig. 2:**
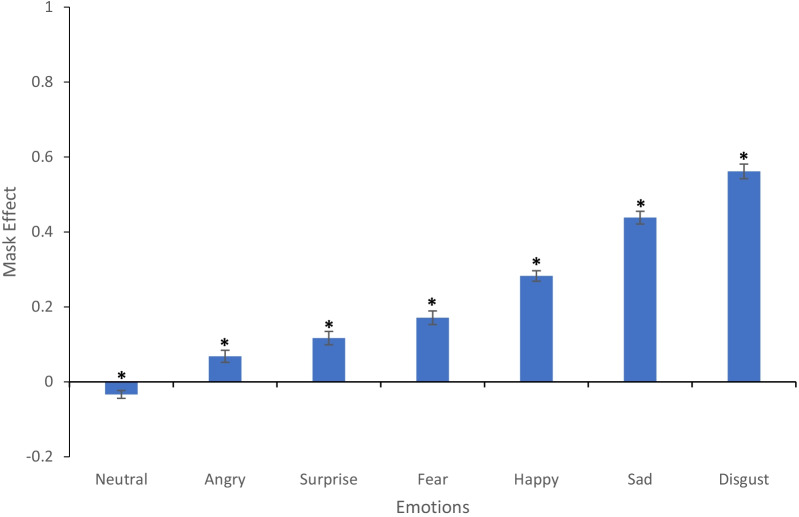
The “mask effect” (i.e. the difference between unmasked and masked emotion recognition accuracy) for each emotion. *Represents a significant difference between performance for masked and unmasked faces, *p* < 0.05. Unmasked vs masked performance for each emotion from the paired samples *t* test

Masks did affect recognition of some emotions more than others (Fig. 2). A repeated measures ANOVA was conducted to explore if the mask effect (calculated by subtracting accuracy for masked faces from accuracy for unmasked faces) for each emotion was different from each other. There was a significant effect of emotion on mask effects, *F* (6, 780) = 173.4, *p* < 0.001, *η*^2^_p_ = 0.57. Post hoc tests (using the Bonferroni correction) were conducted to explore the mask effects. This showed that the mask effect for disgust was significantly larger than the mask effect for all other emotions (all *ps* < 0.001). Similarly, the mask effect for sad faces was significantly larger than all emotions other than disgust (all *ps* < 0.001). The mask effect for neutral was significantly lower compared to all other emotions too (all *ps* < 0.001). There was no significant difference between the size of the mask effect for anger and surprise (*p* = 0.683) and for fear and surprise (*p* = 0.353).

Confusion matrices were created to explore which emotions were confused with other emotions (Table 3). The matrices show that in the masked condition there were many more misclassifications than in the unmasked condition.

**Table 3:** Masked and unmasked stimuli confusion matrix, with mean percentage of each response to each emotion and masked and unmasked negative misattributions to neutral and positive emotions (misattributions of anger, disgust, fear, and sad for happy, surprise, and neutral)

	Masked stimuli								Unmasked stimuli							
	Perceived emotion							Negative misattributions	Perceived emotion							Negative misattributions
	Anger	Disgust	Fear	Neutral	Happy	Sad	Suprise		Anger	Disgust	Fear	Neutral	Happy	Sad	Suprise	
Anger	**68.4%**	10.4%	4.0%*	3.9%*	2.7%	3.8%*	5.9%*		**76.2%**	12.3%	2.5%	1.3%	2.0%	2.4%	2.2	
Disgust	37.8%*	**21.1%**	5.3%*	7.8%*	10.4%*	8.7%*	7.6%*		6.4%	**77.9%**	2.7%	1.7%	5.5%	1.9%	2.9	
Fear	2.0%	3.8%	**28.5%**	4.9%*	2.5%*	3.8	53.8%*		2.2%	12.7%	**46.4%**	1.8%	1.2%	6.4%	28.6	
Neutral	2.3%	1.5%	0.9%	**87.9%**	2.0%	3.9	1.1%	2.1%	2.2%	2.6%	0.7%	**83.9%**	2.2%	5.2%	2.0%	2.7%
Happy	1.5%*	1.4%	1.3%	25.3%*	**65.5%**	2.0%*	2.2%*	1.5%	0.7%	0.7%	0.9%	0.0%	**93.7%**	0.4%	0.8%	0.7%
Sad	10.9%*	12.3%*	9.7%*	28.4%*	1.5%*	**32.4%**	7.8%*		3.9%	8.1%	4.4%	5.9%	0.6%	**77.8%**	2.3%	
Suprise	1.5%	1.7%	11.3%	9.8%*	3.9%	2.5%*	**64.6%**	4.2%	1.0%	1.7%	8.7%	1.3%	5.3%	1.5%	**76.1%**	3.2%

*Represents misclassifications made more frequently in masked conditions, Bonferroni-corrected pairwise comparisons *p* < .05. Bolded numbers represent correct responses

Represents misclassifications made more frequently in unmasked conditions

To explore whether masks altered the pattern of confusions/misclassifications of emotions, seven 2 (mask wearing: masked, unmasked) × 6 (emotion selected) ANOVAs were conducted (one for each of the basic emotions and neutral). For example, the ANOVA on incorrect responses to angry faces examined how often individuals misidentified anger as one of the other five emotions or neutral in both the masked and unmasked conditions. All ANOVAs, except neutral, had a significant interaction between perceived emotion category and mask wearing: angry: *F* (5, 650) = 6.3, *p* < 0.001, *η*^2^_p_ = 0.05, disgust: *F* (5, 650) = 94.2, *p* < 0.001, *η*^2^_p_ = 0.42, fear: *F* (5, 650) = 135.3, *p* < 0.001, *η*^2^_p_ = 0.51, neutral: *F* (5, 650) = 1.6, *p* = 0.158, *η*^2^_p_ = 0.01, happy: *F* (5, 650) = 208.3, *p* < 0.001, *η*^2^_p_ = 0.62, sad: *F* (5, 650) = 53.2, *p* < 0.001, *η*^2^_p_ = 0.29, surprise: *F* (5, 650) = 19.5, *p* < 0.001, *η*^2^_p_ = 0.13.

As a result of these significant interactions, pairwise comparisons (with Bonferroni correction) were conducted to examine which misclassifications occurred more in masked than unmasked conditions. Significant differences in misclassifications are indicated in the confusion matrices (Table 3). Notably, fear was misclassified as surprise in over half of the trials in the masked condition (53.8%). Also in the masked condition, disgust was more frequently misclassified as anger, and happiness and sadness were more frequently misclassified as neutral expressions. Unexpectedly, fear was misclassified as disgust and sadness more frequently in unmasked than in masked conditions. Also, neutral was misclassified as disgust and surprise more frequently in unmasked conditions.

To answer the question of whether masks created similar misattributions as religious coverings, as explored in Kret and De Gelder (2012), we investigated whether neutral and positive emotions in the masked condition were misattributed with negative emotions more frequently than unmasked faces. A 2 (mask wearing: masked, unmasked) × 3 (emotions: happy, surprise, neutral) repeated measures ANOVA was conducted. The dependent variable, negative misattributions, was the proportion of the anger, fear, disgust, and sad misattributions for the emotions of happy, surprise, and neutral. There was a significant main effect of mask, *F* (1, 130) = 6.9, *p* = 0.010, *η*^2^_p_ = 0.05, and emotions, *F* (2, 260) = 57.5, *p* < 0.001, *η*^2^_p_ = 0.31. There was a significant interaction between masks and emotion, *F* (2, 260) = 9.5, *p* < 0.001, *η*^2^_p_ = 0.07.

Post hoc comparisons revealed a significant difference between masked and unmasked faces, *t* (130) = 2.6, *p* = 0.010). Masked faces (*M* = 0.03) had a greater number of negative misattributions than unmasked faces (*M* = 0.02). There were significant differences between all emotions (all *p*s < 0.001), with the greatest number of misattributions for surprise (*M* = 0.04), followed by neutral (*M* = 0.02) and happy (*M* = 0.01). Post hoc comparisons of masks and emotions show significant differences between all conditions except for masked happy and masked neutral (*p* = 0.110), masked surprise and unmasked surprise (*p* = 0.127), and masked neutral and unmasked neutral (*p* = 0.491).

In summary, the results indicate that masks have a significant negative impact on emotion recognition. However, the extent of this impact varies across emotions, with neutral being unaffected and disgust being most affected. The results also show how masks can cause confusion in emotion recognition. The most frequent confusions observed were fear with surprise in masked and unmasked conditions, disgust with anger in masked conditions, and happy and sad with neutral expressions in masked conditions.

### Masks and in-group effects

Previous research suggests that participants are better at recognising emotions in individuals who are the same ethnicity as them (Elfenbein & Ambady, 2002). As such, this analysis investigated whether the match (or mismatch) of stimulus and perceiver ethnicity affected emotion recognition accuracy with and without masks. To investigate any differences in performance based on ethnicity, the stimuli were split into three groups based on ethnicity (Asian, Black, White). A linear mixed effects model was employed to explore mask condition (masked, unmasked) and participant ethnicity (same as stimuli, different to stimuli) for each of the stimulus sets. Using the Asian stimuli as an example, participants were categorised as either Asian observers, or non-Asian observers (i.e. the non-Asian group contained all participants that were not Asian). The fixed factors were mask condition, stimuli ethnicity (Asian or non-Asian), and participant ethnicity (Asian or non-Asian), and the random variables were participant and actor. This was then repeated for White and Black stimuli, with participants classified accordingly. We were specifically interested in (1) whether there was a main effect of participant ethnicity (suggesting an own-ethnicity effect for emotion recognition); and (2) whether ethnicity interacted with mask wearing (suggesting that the effects of mask wearing on emotion accuracy might be modulated by the ethnicity of the mask wearer and the perceiver).

### Asian stimuli

A significant main effect of mask wearing was found, *F* (24.5) = 211.4, *p* < 0.001. No significant effect of participant ethnicity, *F* (28.5) < 0.1, *p* = 0.978, or stimuli ethnicity, *F* (12.9) = 0.5, *p* = 0.477, was found. There was no significant interaction between mask wearing, participant ethnicity, and stimuli ethnicity found, *F* (20,215.2) = 0.6, *p* = 0.424. The variance of participant was < 0.01 (ICC < 0.001) and the variance of actor was < 0.01 (ICC = 0.009), both small. Actor and participant variance in the mask condition, stimuli ethnicity, and participant ethnicity were all small (ranging from < 0.01 to 0.04). Fixed effects parameter estimates are presented in Table 4. A complete table with fixed effects and random components is found in Appendix 5.

**Table 4:** Fixed effects table for the Asian ethnicity linear mixed effects model

Fixed effects	Estimate	Standard error	95% CI	*t*	*p*
Mask condition (unmasked–masked)	0.23	0.02	0.20, 0.26	14.5	< 0.001
Stimuli ethnicity (Asian–non-Asian)	0.03	0.04	− 0.04, 0.09	0.7	0.477
Participant ethnicity (Asian–non-Asian)	− < 0.01	0.02	− 0.04, 0.04	< 0.1	0.978
Mask * stimuli ethnicity	− 0.02	0.03	− 0.08, − 0.05	− 0.6	0.586
Mask * participant ethnicity	− < 0.01	0.02	− 0.03, 0.03	< 0.1	0.963
Stimuli ethnicity * participant ethnicity	0.01	0.02	− 0.02, 0.04	0.5	0.642
Mask * stimuli ethnicity * participant ethnicity	− 0.02	− 0.03	− 0.08, 0.03	− 0.8	− 0.424

### Black stimuli

A significant main effect of mask wearing was found, *F* (30.3) = 186.1, *p* < 0.001. No significant effect of participant ethnicity, *F* (31.2) = 1.0, *p* = 0.337, or stimuli ethnicity, *F* (13.4) = 2.5, *p* = 0.134, was found. There was no significant interaction between mask wearing, participant ethnicity, and stimuli ethnicity found, *F* (21,023.2) < 0.1, *p* = 0.882. The variance of participant was 0.01 (ICC = 0.056) and actor was < 0.001, (ICC < 0.001). Actor and participant variance in the mask condition, stimuli ethnicity, and participant ethnicity were all small (ranging from < 0.01 to 0.02). Fixed effects parameter estimates are presented in Table 5. A complete table with fixed effects and random components is found in Appendix 6.

**Table 5:** Fixed effects table for the black ethnicity linear mixed effects model

Fixed effects	Estimate	Standard error	95% CI	*t*	*p*
Mask condition (unmasked–masked)	0.23	0.02	0.20, 0.26	13.6	< 0.001
Stimuli ethnicity (black–non-black)	− 0.05	0.03	− 0.10, 0.01	− 1.6	0.134
Participant ethnicity (black–non-black)	− 0.03	0.03	− 0.09, 0.03	− 1.0	0.337
Mask * stimuli ethnicity	0.02	0.03	− 0.04, 0.09	0.7	0.472
Mask * participant ethnicity	− 0.03	0.02	− 0.07, 0.01	− 1.4	0.157
Stimuli ethnicity * participant ethnicity	− 0.1	0.02	− 0.04, 0.03	− 0.3	0.789
Mask * stimuli ethnicity * participant ethnicity	− 0.1	0.04	− 0.07, 0.06	− 0.1	0.424

### White stimuli

A significant main effect of mask wearing was found, *F* (25.8) = 209.6, *p* < 0.001. No significant effect of participant ethnicity, *F* (54.7) = 0.1, *p* = 0.724, or stimuli ethnicity, *F* (20.3) = 0.6, *p* = 0.431, was found. There was no significant interaction between mask wearing, participant ethnicity, and stimuli ethnicity found, *F* (21,159.1) = 0.7, *p* = 0.396. The variance of participant was 0.01 (ICC = 0.057) and actor was < 0.01, (ICC = 0.006). Actor and participant variance in the mask condition, stimuli ethnicity, and participant ethnicity were all small (ranging from < 0.01 to 0.01). Fixed effects parameter estimates are presented in Table 6. A complete table with fixed effects and random components is found in Appendix 7.

**Table 6:** Fixed effects table for the white ethnicity linear mixed effects model

Fixed effects	Estimate	Standard error	95% CI	*t*	*p*
Mask condition (unmasked–masked)	0.23	0.02	0.20, 0.26	14.5	< 0.001
Stimuli ethnicity (white–non-white)	0.02	0.03	− 0.03, 0.07	0.8	0.431
Participant ethnicity (white–non-white)	0.01	0.02	− 0.04, 0.06	0.4	0.724
Mask * stimuli ethnicity	− 0.02	0.03	− 0.08, 0.04	− 0.6	0.573
Mask * participant ethnicity	− < 0.1	0.02	− 0.03, 0.03	< 0.1	0.964
Stimuli ethnicity * participant ethnicity	− 0.01	0.01	− 0.03, 0.02	− 0.3	0.755
Mask * stimuli ethnicity * participant ethnicity	− 0.02	0.03	− 0.08, 0.03	− 0.8	0.396

**Fig. 3:**
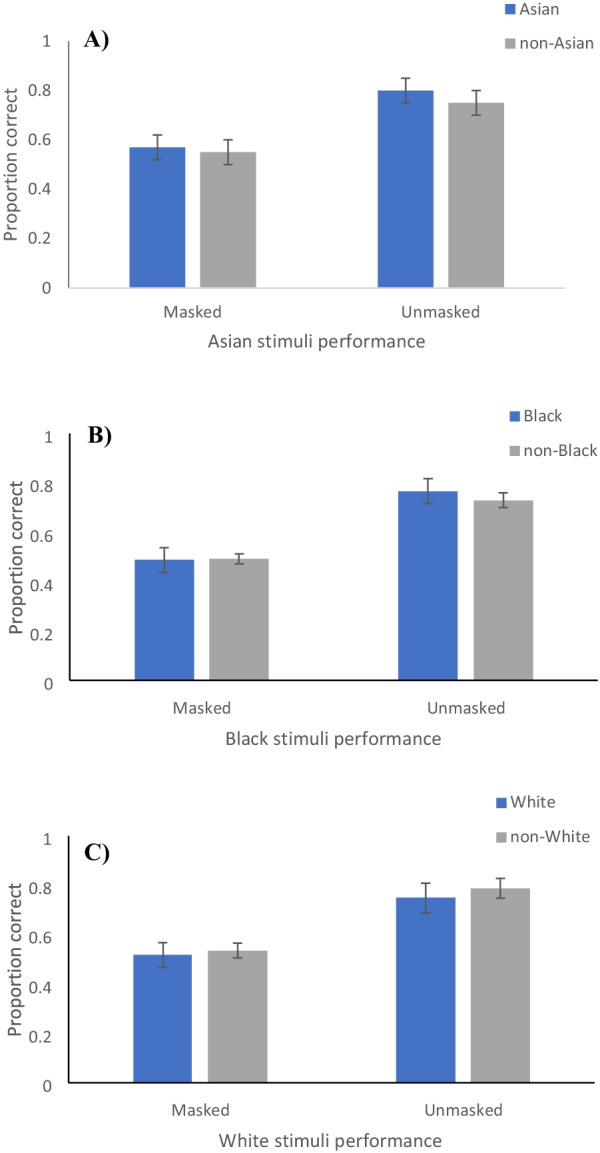
Bar graphs showing the proportion correct for masked and unmasked Asian, Black, and White stimuli: **A** Asian faces, split by Asian vs non-Asian observers. **B** Black faces, split by Black vs non-Black observers. **C** White faces, split by White vs non-White observers. Error bars represent 95% confidence intervals

The results, for all ethnicities, show there was a significant effect of masks, but not participant ethnicity, and no significant interaction (Fig. 3). This reinforced our previous conclusion that masks reduce emotion recognition accuracy. However, there was no evidence that the effect of masks on emotion recognition is modulated by the match (or mismatch) between perceiver and mask-wearer ethnicity. In sum, we did not find an own ethnicity, or in-group, effect on emotion recognition overall.

### Attitudes towards masks

To understand the effect of attitudes on masked and unmasked emotion recognition accuracy, the responses from the mask-wearing attitudes questionnaire were entered into a principal components analysis (PCA). Due to high correlations (*r*’s > 0.8) between items 1a and 1b, 2a and 2b, and 3a and 3b (mask wearing and attitudes towards individuals who do/do not wear masks on public transport and in shops), responses across those items were averaged. This resulted in three new items: mask wearing on public transport AND in shops, subsequently 1e; attitudes towards people who wear masks on public transport AND in shops, subsequently 2e; and attitudes towards people who do not wear masks in public transport AND in shops, subsequently 3e. An initial PCA revealed very low communality for the item relating to reasons for not wearing masks (*h*^2^ = 0.19), so this item was excluded from the final PCA.

A PCA with oblique rotation (direct oblimin) was carried out on the remaining 9 items. Bartlett’s test of sphericity was significant (*χ*^2^ (36) = 756, *p* < 0.0005); the Kaiser–Meyer–Olkin measure of sampling adequacy was acceptable (KMO = 0.79); and all KMO values for individual variables were above 0.76 (above the acceptable limit of 0.5, Field, 2018). This confirms the data were suitable for PCA. An inspection of the scree plot and components with eigenvalues greater than 1 confirmed that a two-component solution was appropriate for the data. Table 7 shows the pattern matrix following rotation for the final component solution (component loadings lower than 0.3 are omitted). The items that load onto each factor suggest that a high score on the first component (Inside) represents a tendency to wear masks more frequently inside (transport/shops and other indoor situations). Higher scores on the first factor also reflect a tendency to rate individuals who wear masks inside (and to a lesser extent, outside) more positively, and those who do not wear masks inside more negatively.

**Table 7:** Rotated component loadings for each item in the attitude questionnaire

Item	Rotated component loadings	
	Component 1 (Inside)	Component 2 (Outside)
2c: Rating of someone wearing a face covering inside	0.92	
2e: Rating of someone wearing a face covering on public transport AND in shops/businesses	0.87	
1e: Likelihood of wearing a face covering on public transport AND in shops/businesses	0.76	
1c: Likelihood of wearing a face covering inside	0.62	
3e: Rating of someone NOT wearing a face covering on public transport AND in shops/businesses	− 0.59	
3c: Rating of someone NOT wearing a face covering inside	− 0.56	− 0.35
3d: Rating of someone NOT wearing a face covering outside		− 0.91
1d: Likelihood of wearing a face covering outside		0.57
2d: Rating of someone wearing a face covering outside	0.36	0.45

At the time of data collection (Jan–Feb 2021), face coverings were a legal requirement in most inside environments in the UK (but generally not outside). Therefore, this component may reflect a higher tendency to obey rules around face coverings and to look positively on those who also obey those rules (and negatively on those who do not). The second component primarily reflects responses to questions about outside mask wearing. Higher scores on the second component reflect more positive attitudes towards those who wear masks Outside, negative attitudes towards those who do not wear masks Outside, and a higher likelihood of outside mask wearing. Higher scores on this item also reflect a slightly more negative rating of individuals who do not wear masks in indoor environments. We interpret this component as broadly reflecting negative attitudes towards the use of face coverings in environments where they are not required.

**Fig. 4:**
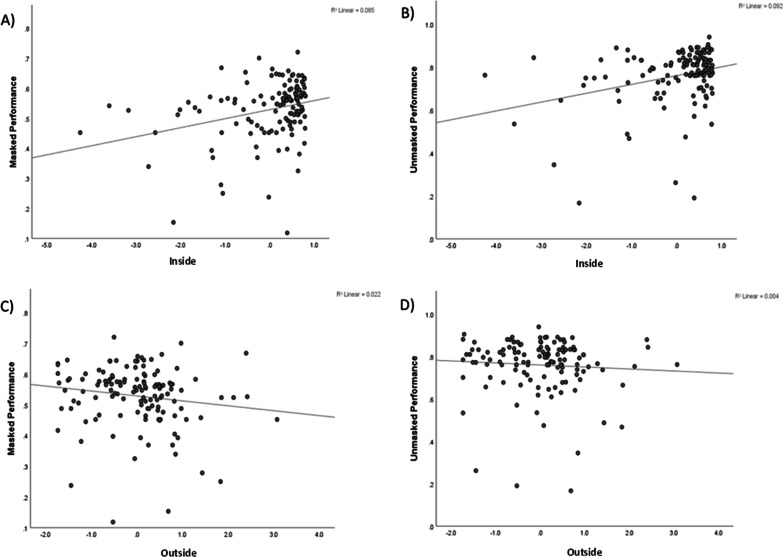
Scatter graphs showing the correlation between the components and masked or unmasked emotion recognition performance: **A** Inside and masked performance. **B** Inside and unmasked performance. **C** Outside and masked performance. **D** Outside and unmasked performance

Spearman’s rho was employed to assess the correlation between attitudes towards mask wearing Inside and Outside and the performances for both masked and unmasked conditions (Fig. 4). There was a significant correlation between Inside and masked performance, *r*_s_ (131) = 0.33, *p* < 0.001, 95% CI [0.16, 0.48], Inside and unmasked performance, *r*_s_ (131) = 0.26, *p* = 0.003, 95% CI [0.09, 0.42], and Outside and masked performance, *r*_s_ (131) = 0.24, *p* = 0.005, 95% CI [- 0.07, 0.40]. There was not a significant correlation between Outside and unmasked performance, *r*_s_ (131) = 0.16, *p* = 0.072, 95% CI [- 0.01, 0.32].

Fisher’s Z-transformation was used to assess whether the correlations between the components and masked and unmasked performance differed significantly. There was not a significant difference between the correlations for masked and unmasked performance and component 1 (Inside), *z* = 1.03, *p* = 0.152, or between the correlations for masked and unmasked performance and component 2 (Outside), *z* = 1.14, *p* = 0.127. This suggests that for each of the components, the correlations with masked and unmasked performances were similar.

To further explore the misattribution of negative emotions to positive and neutral emotions, we performed a Spearman’s rho correlation. There were no significant correlations between attitudes towards masks Inside and masked, *r*_s_ (131) =  − 0.08, *p* = 0.358, and unmasked negative misattributions, *r*_s_ (131) =  − 0.02, *p* = 0.824, or between attitudes towards masks Outside and masked, *r*_s_ (131) = 0.04, *p* = 0.678, and unmasked negative misattributions, *r*_s_ (131) = 0.09, *p* = 0.305. We found no evidence that attitudes towards masks correlated with a greater attribution of negative emotions to masked faces.

In summary, attitudes did have some relationship with emotion recognition. Attitudes towards masks Inside were associated with emotion recognition overall for masked and unmasked faces, whereas attitudes towards masks Outside were only associated with emotion recognition for masked faces. This suggests that negative attitudes towards Outside mask wearing predict poorer performance with masked faces but do not predict emotion recognition more generally.

The data sets used and analysed are available on the Open Science Framework repository (https://osf.io/57nfe/).

## Discussion

The main aims for this study were to investigate the effects of masks on emotion recognition and how this may vary depending on participant and stimulus ethnicity, and attitudes towards mask wearing. In line with previous research (e.g. Marini et al., 2021; Noyes et al., 2021), our data indicate that masks have a significant effect on emotion recognition. Emotion recognition for masked faces was significantly poorer compared to unmasked faces. This effect was particularly pronounced for disgust, sad, and happy expressions.

In addition to replicating and extending on previous findings (Carbon, 2020), we present novel findings regarding individual differences which affect masked and unmasked emotion recognition. No evidence of an “other ethnicity effect” was found, regardless of whether the faces were masked or not. Further, there was no evidence that the effects masks have vary depending on whether participants were viewing faces of the same or different ethnicities to themselves.

However, the present findings show that mask attitudes did correlate with emotion recognition performance. Attitudes towards mask wearing Inside (component 1) correlated with both masked and unmasked emotion recognition performance. Attitudes towards mask wearing Outside (component 2) correlated with masked emotion recognition, but not unmasked emotion recognition.

### Masks and emotion recognition

There was an association between masks and poorer emotion recognition accuracy. Our results are consistent with previous research which found that face coverings lead to poorer emotion recognition overall (Marini et al., 2021; Noyes et al., 2021). There is previous research concluding a minimal impact of masks on social interactions (Ruba & Pollak, 2020). This differs from our conclusions, and previous studies. The difference in conclusions could be due to the different age groups tested or the edited masks not covering the same areas as real-life mask wearing. Most likely, the difference is due to the editing of the masks (or other task-related factors), as previous research investigating a younger sample found similar results to ours that masks affected emotion recognition (Gori et al., 2021).

Masks not only affected overall recognition but affected specific emotions too. This is not surprising: previous research using partially occluded faces supports our mask effect findings. Research found certain emotions are recognised using the lower face region (disgust and happiness) (Bassili, 1979; Wegrzyn et al., 2017), which suggests that the effects of masks may vary depending on the emotion being expressed. As would be predicted from partial face studies, we found that masked disgusted faces had the lowest accuracy, and the largest accuracy difference between the masked and unmasked conditions. However, contrary to what might be expected from partial face work (Eisenbarth & Alpers, 2011; Wegrzyn et al., 2017), our results indicate that accuracy for masked happy faces remained relatively high. Previous research has found happiness was the easiest to recognise when masked, even though it relies on the lower region of the face. Grahlow et al. (2021) found anger, sadness, and disgust were most affected by masks, but not fear, happy, or neutral. This is consistent with the current study, as happiness recognition was not most affected by masks. Also, consistent with the current study, Grahlow et al. (2021) found the neutral faces were least effected by masks, while sadness and disgust were most affected. A possible explanation for why our happiness recognition differed from partial face research is the intensity of the expressions in the current stimuli. The database used in the current study did not provide intensity ratings, so the stimuli we used may have included more intense “happy” expressions than previous research, leading to more accurate emotion recognition. Future research should aim to include emotional stimuli varying in intensity to explore if the effects of masks on emotion recognition accuracy vary depending on intensity.

Similar to previous work (Marini et al., 2021; Noyes et al., 2021), we found neutral recognition accuracy was not negatively affected by masks. In fact, we found that neutral recognition was actually higher in masked than unmasked conditions. This could be because masks introduced a bias towards “neutral” responses. Our confusion matrices show higher neutral misclassifications for every emotion in the masked condition; emotional faces were mislabelled with neutral significantly more often when masked. It is possible the neutral responses were higher in masked conditions because it acted as a “default” response if participants were unsure what emotion was being expressed. Alternatively, given that many emotional cues are carried in the lower half of the face (Bassili, 1979; Wegrzyn et al., 2017), participants may have perceived masked faces as unemotional more of the time. Due to the simple forced-choice paradigm that was employed in the current study, our findings cannot discriminate between these two explanations. While forced-choice responses have the advantage of being simple to understand (which was a priority for us given the online nature of the work), this method can exaggerate recognition scores and measure decision strategies, such as process of elimination, instead of pure recognition (Nelson & Russell, 2013). Consequently, future work should consider the use of open-ended responses or an “I don’t know” and “other” response option (with the option to free label).

Another notable finding was that fear recognition in both conditions was relatively low. Our findings of fear having poor recognition in both conditions differ from previous research. Contrasting Carbon (2020) and Bani et al.’s (2021) results, of fear not being affected by face masks, we found a significant decrease in accuracy across all emotions, except neutral. Fear had the lowest recognition accuracy in unmasked condition and the second lowest in masked condition. Our unmasked fear accuracy was low in comparison with Carbon (2020). This is unexpected as fear is classed as “threat recognition” which can be recognised from presentations of 39 ms (Bar et al., 2006). An explanation is that fear recognition was lower because it was misclassified as surprise in both masked (53.8% of trials) and unmasked (28.6% of trials) conditions. Carbon (2020) excluded surprise, hence why these findings differ. Fear and surprise share similar perceptual characteristics; both are expressed with wide eyes and raised eyebrows (Sacco & Hugenberg, 2009). Research using isolated eyes (Chamberland et al., 2017), unmasked faces presented rapidly (Zhao et al., 2017), and a younger sample (Ruba & Pollak, 2020) have supported the frequent confusion between fear and surprise. This indicates that the effects observed in our study for these specific emotions likely arise due to perceptual occlusion, as opposed to specific social biases associated with masks.

Masks also changed the pattern of misclassifications that occurred when emotions were incorrectly identified. Our misclassification findings are supported by previous research. We found that disgust was misclassified as anger in 37.8% of cases in masked conditions, consistent with Carbon (2020). Although our biggest misclassification was fear with surprise, not disgust with anger, misclassifications could potentially pose problems with acting appropriately in social situations as different emotions prompt different actions. One possible explanation for the disgust with anger confusion would be the similar perceptual characteristics of the two emotions. Both are expressed by lowered eyebrows (Sacco & Hugenberg, 2009), but the vital differences lie in the lower region of the face and are not visible in the masked condition. For anger, the lips are pressed together, whereas for disgust the upper lip is raised and the lower lip is protruding (Ekman & Friesen, 2003). Also, in line with the evolutionary perspective, detection of both anger and disgust serves a similar purpose—promoting avoidance of dangerous situations. Given their importance to behaviour, the detection of these emotions should be relatively robust to suboptimal viewing conditions; however, there is little pressure to make more intricate discriminations.

The current findings support the contention that masks could impact everyday social interactions. While this study focused on emotion recognition, other studies suggest that other aspects of social interaction might also be impaired by mask wearing. Saunders et al. (2021) explored the impact of face coverings on hearing and communication. They found that face coverings negatively affected hearing, understanding, engagement, and feelings of connection with the speaker. Alongside this, masks also increased anxiety and stress and made communications tiring and frustrating. Those with hearing loss were significantly more impacted. These results show the wider impact of masks on communication. The results support the importance of “communication-friendly” masks. This is similar to Marini et al.’s (2021) suggestion of transparent masks. These results also raise the question of whether the effects on emotion recognition could be minimised with more naturalistic stimuli. While previous research largely agrees that masks negatively affect emotion recognition, regardless of whether the stimuli is naturalistic or edited faces, future research may consider the use of other naturalistic stimuli (e.g. moving stimuli) to address this question.

### In-group bias and emotion recognition

This study found no significant effects of own-ethnicity bias on emotion recognition. Specifically, emotion recognition performance and the effects of masks on emotion recognition performance did not differ regardless of whether there was a match or mismatch between participant and stimulus ethnicity. This is unexpected, as a cross-cultural meta-analysis (Elfenbein & Ambady, 2002) found that recognition accuracy was higher when viewing members of the same ethnicity. However, the meta-analysis explicitly noted that in-group ethnicity effects were weakened in more culturally diverse populations. The current study was conducted in London (UK) employing a sample of university students with extensive exposure to different ethnicities. A limitation of the current study is that information regarding participant’s experience and exposure with other ethnicities was not collected. Research suggests that childhood experience plays an important role in subsequent processing of other-ethnicity faces (McKone et al., 2019) and as a result of recruiting from a London-based University, the participants would have extensive experience with White faces. Nonetheless, our findings do not align with the social motivation theory (Young & Hugenberg, 2010) as we did not find in-group (own ethnicity) stimuli resulted in more accurate face processing compared to out-group (other ethnicity) stimuli as suggested.

Even though ethnicity did not appear to act as a social cue which impacted emotion recognition, it is possible that masks did. Previous research has established that face coverings (e.g. religious face coverings) might act as a social grouping cue (thereby impacting emotion recognition), regardless of ethnicity (Kret & De Gelder, 2012). Furthermore, previous work has found that social grouping cues can override ethnicity effects in face recognition (Van Bavel & Cunningham, 2012). It is theoretically possible that the face masks used in the current study had a similar effect. This could potentially explain the absence of own-ethnicity biases in our data, and the pattern of errors that participants made for masked faces. In line with the previous religious coverings research (Kret & De Gelder, 2012), we found that participants made more negative misattributions to masked faces than unmasked faces. This raises the possibility that masks were acting as a social grouping cue and affecting emotion attribution in a similar manner. However, the vast majority of our sample reported high levels of face mask use which makes it less likely that mask wearers would be considered an “out-group” for most of our participants. Given the very small numbers of individuals who reported low mask usage in the current sample, it is not possible to compare the effects of mask wearing (and, by extension, “in-groups” and “out-groups” based on mask wearing) on emotion effects in more depth. However, future studies may wish to selectively recruit individuals with different patterns of mask-wearing behaviour to examine potential in-group and out-group biases (and their interaction with other social cues such as ethnicity) in more depth.

These results have important practical implications. They indicate that it is unlikely the effects of masks on emotion recognition vary dramatically as a result of social grouping cues like ethnicity. Instead, it is more likely that, for most individuals, the negative effects of masks on emotion recognition are quite consistent across the faces being observed and are likely attributable to perceptual occlusion. As such, alternative protective measures which focus on improving the availability of perceptual information (e.g. transparent masks; Marini et al., 2021; or face shields) are likely to be widely effective at minimising emotion recognition difficulties.

### Mask attitudes and emotion recognition

The majority of our sample reported frequent mask wearing in indoor environments: 80.2% selected 7 (always) in response to “How likely are you to wear a face covering on public transport”; and 72.5 and 60.3% reported always wearing a mask inside shops/businesses and other indoor environments, respectively. This is in line with previous research reporting similar distributions (Taylor & Asmundson, 2021). The present findings found that certain attitudes—specifically, attitudes towards masks Inside—were associated with emotion recognition accuracy in masked and unmasked faces. This seems to reflect broader emotion recognition abilities. On the other hand, negative attitudes towards masks Outside were associated with poorer masked emotion recognition. It seemed initially that attitudes towards masks Outside may be associated specifically with masked emotion recognition, rather than overall emotion recognition. However, as the correlations were not significantly different, we cannot draw any strong conclusions about whether this association is specific or whether it may also reflect broader emotion recognition abilities. While there is evidence for an association between overall emotion accuracy and attitudes, we did not find an association between attitudes towards masks and misattribution of negative emotions to masked and unmasked faces. Negative attitudes towards outside mask wearing being associated with poorer masked emotion recognition, but not specifically negative misattributions, could be due to factors such as reduced attention or lack of perceptual expertise to masked faces.

While there is some evidence that mask attitudes can affect judgements of trustworthiness (Biermann et al., 2021), to date, mask attitudes have not been investigated in terms of emotion recognition. Research exploring attitudes towards different ethnicities and political attitudes (Fischer et al., 2012; Van Hiel et al., 2019) found that attitudes can influence emotion recognition. Previously discussed research found a significant effect of participant attitudes. Van Hiel et al. (2019) found that right-wing political attitudes had a negative effect on emotional abilities. These results are in line with our current findings of attitudes being significant. This suggests that attitudes towards different groups/characteristics, or attitudes more generally, can affect emotion recognition ability.

It is possible that some of the association between positive attitudes towards masks and better emotion recognition performance with masked faces may be driven by the mere exposure effect (Fang et al., 2007). The mere exposure effect is when repeated exposure leads to an increased liking. For example, if individuals spend more time around people who wear masks, then they might be more likely to develop a positive attitude towards masks. Increased exposure to masked faces may also improve emotion recognition by increasing perceptual expertise. However, there is evidence for the effect of masks on other face processing tasks staying consistent over 13 months of the pandemic (Bennetts et al., 2022). Previous research has found that perceptual learning can shape emotion perception (Pollak et al., 2009). Therefore, it is possible that people could develop a perceptual expertise for masked faces and this may be associated with improved emotion recognition accuracy for masked faces. Perceptual expertise might also explain the own-ethnicity effect results. Exposure to other ethnicities would increase perceptual expertise which could explain the improved accuracy for other ethnicities and therefore the lack of own-ethnicity effect found.

Another possible explanation for why positive mask attitudes Inside (which also reflected higher mask wearing Inside) were associated with better emotion recognition generally may be because those who wear masks more diligently completed the task more diligently. Previous research found COVID-19 precautionary behaviours were most consistently related to higher conscientiousness, neuroticism, and openness (Airaksinen et al., 2021). This suggests that those more conscientious, who would complete tasks with more care, seem to also take precautions with COVID-19, which would include mask wearing.

The attitude results—specifically, attitudes towards masks Inside—could also be linked to empathy. Higher levels of empathy are associated with better emotion recognition abilities (Besel & Yuille, 2010; Israelashvili et al., 2020; Wearne et al., 2020). Therefore, better overall emotion recognition performance on the task may reflect higher levels of empathy. If this is the case, the relationship between attitudes and emotion recognition performance supports previous explanations. For example, the empathy–attitude effect (Batson et al., 2002) suggests that there is a close association between the two concepts: you tend to empathise with other people who share the same views as you, and empathy improves attitudes even when the person who induced the empathy is not prototypical of the group.

Due to the correlational nature of our study, we cannot be sure if attitudes lead to worse masked emotion recognition or if worse emotion recognition leads to negative attitudes. Depending on the causal direction, this has potential implications for reducing the negative effects of masks on emotion recognition by attempting to improve attitudes towards masks/empathy towards mask wearers. For example, our findings suggest that negative attitudes towards masks may be associated with poorer masked emotion recognition particularly when masks are deemed unnecessary (not mandatory) in line with government guidelines. This could suggest that, when discussing masks and mask mandates, the phrasing could be amended to emphasise they are recommended in all situations where social distancing is not possible, even if they are not formally required. However, we cannot be certain about the direction of the attitude–emotion recognition effects, or the relationship between the attitude scale and broader constructs (e.g. empathy, specific difficulties experienced with masks). Therefore, further examination of factors that correlate with and contribute to mask attitudes is needed.

## Conclusion

To conclude, this study explored the effects of face masks on emotion recognition, and how these effects can be modulated by individual differences (in-group biases and mask attitudes). We found that masks were negatively associated with emotion recognition accuracy and also increased confusion of certain emotions with others. This suggests that masks can have wide-ranging implications on interpersonal interactions. There was no in-group advantage (when exploring an ethnicity match and mismatch) observed overall or in interaction with masks. However, even though ethnicity did not show an in-group advantage, masks may have acted as a social cue. Attitudes towards masks correlated with masked and unmasked emotion recognition. Attitudes towards mask wearing Inside correlated with emotion recognition generally and attitudes towards mask wearing Outside correlated with masked emotion recognition only. This suggests that negative attitudes towards mask wearing Outside (where not mandatory) could potentially lead to worse emotion recognition for masked faces, although the causal direction of the effect is unclear. More research is needed on whether the effects on emotion recognition are consistent across more naturalistic stimuli (e.g. moving stimuli) and how to overcome the mask effects (e.g. modified masks). As we found limited interactions between social cues (e.g. own-ethnicity bias) and mask wearing, we tentatively conclude that the negative effects of masks are primarily perceptual in nature; however, these effects may be modulated by mask attitudes.

## Data Availability

As noted in the paper, the datasets generated and analysed during the current study are available in the Open Science Framework repository (https://osf.io/57nfe/). A fully programmed version of the experiment is available upon request from the authors.

## Appendix 2: The average accuracy of the identities

**Table:**

Identity	Average accuracy
Asian	84.69642857
AF02	95.28571429
AF07	90.57142857
AF09	83.28571429
AF11	86.14285714
AM04	84.28571429
AM05	83.71428571
AM06	78.42857143
AM11	75.85714286
Black	80.01785714
BF05	83.42857143
BF07	78.57142857
BF15	80.85714286
BF16	72.28571429
BM07	84.28571429
BM12	71.42857143
BM16	87.42857143
BM17	81.85714286
White	84.03636364
WF03	79.71428571
WF06	86
WF09	76.14285714
WF14	87.42857143
WM01	87.66666667
WM04	84.14285714
WM07	85.71428571
WM09	86
Grand total	82.91017964

## Appendix 3: Attitudes towards masks questionnaire

How likely are you to wear a face covering on public transport (e.g. trains, buses, underground/tube)?

How likely are you to wear a face covering inside shops/businesses (when not eating or drinking)?

How likely are you to wear a face covering in other enclosed environments with multiple people inside (e.g. lecture halls)?

How likely are you to wear a face covering when you are outside (e.g. walking down the street)?

What's your most common reason for NOT wearing a face covering?

How would you rate someone who is WEARING a face covering on public transport (e.g. trains, buses, underground/tube)?

How would you rate someone who is WEARING a face covering inside shops/businesses (when not eating or drinking)?

How would you rate someone who is WEARING a face covering in other enclosed environments with multiple people inside (e.g. lecture halls)?

How would you rate someone who is WEARING a face covering outside (e.g. walking down the street)?

How would you rate someone who is NOT WEARING a face covering on public transport (e.g. trains, buses, underground/tube)?

How would you rate someone who is NOT WEARING a face covering inside shops/businesses (when not eating or drinking)?

How would you rate someone who is NOT WEARING a face covering in other enclosed environments with multiple people inside (e.g. lecture halls)?

How would you rate someone who is NOT WEARING a face covering outside (e.g. walking down the street)?

## Appendix 4: Fixed and random effects table for the mask and emotion linear mixed effects model

**Table:**

Fixed effects	Estimate	Standard error	95% CI	*t*	*p*
Mask condition (unmasked–masked)	0.23	0.02	0.21, 0.26	15.7	< 0.001
Emotion 1 (anger–neutral)	− 0.14	0.04	− 0.21, − 0.06	− 3.7	0.001
Emotion 2 (disgust–neutral)	− 0.37	0.04	− 0.44, − 0.30	− 10.5	< 0.001
Emotion 3 (fear–neutral)	− 0.49	0.04	− 0.57, − 0.41	− 12.2	< 0.001
Emotion 4 (happy–neutral)	− 0.07	0.04	− 0.15, 0.02	− 1.5	0.139
Emotion 5 (sad–neutral)	− 0.33	0.04	− 0.41, − 0.26	− 8.8	< 0.001
Emotion 6 (surprise–neutral)	− 0.13	0.03	− 0.19, − 0.06	− 3.8	< 0.001
Mask condition * emotion 1	0.12	0.02	0.08, 0.16	6.3	< 0.001
Mask condition * emotion 2	0.60	0.02	0.57, 0.64	31.9	< 0.001
Mask condition * emotion 3	0.22	0.02	0.18, 0.25	11.3	< 0.001
Mask condition * emotion 4	0.32	0.02	0.28, 0.36	16.9	< 0.001
Mask condition * emotion 5	0.48	0.02	0.44, 0.51	25.4	< 0.001
Mask condition * emotion 6	0.15	0.02	0.11, 0.19	7.9	< 0.001

Random effects	Variance	SD
Participant (Intercept)	0.01	0.10
Mask condition (unmasked–masked)	< 0.01	0.04
Participant| emotion 1 (anger–neutral)	0.02	0.14
Participant| emotion 2 (disgust–neutral)	0.01	0.10
Participant| emotion 3 (fear–neutral)	0.03	0.18
Participant| emotion 4 (happy–neutral)	0.01	0.09
Participant| emotion 5 (sad–neutral)	0.02	0.13
Participant| emotion 6 (surprise–neutral)	0.01	0.09
Actor (intercept) mask condition	0.01 < 0.01	0.07 0.07
Actor| emotion 1 (anger–neutral)	0.03	0.17
Actor| emotion 2 (disgust–neutral)	0.03	0.16
Actor| emotion 3 (fear–neutral)	0.03	0.18
Actor| emotion 4 (happy–neutral)	0.04	0.20
Actor| emotion 5 (sad–neutral)	0.03	0.17
Actor| emotion 6 (surprise–neutral)	0.02	0.15

## Appendix 5: Fixed and random effects table for the Asian ethnicity linear mixed effects model

**Table:**

Fixed effects	Estimate	Standard error	95% CI	*t*	*p*
Mask condition (unmasked–masked)	0.23	0.02	0.20, 0.26	14.5	< 0.001
Stimuli ethnicity (Asian–non-Asian)	0.03	0.04	− 0.04, 0.09	0.7	0.477
Participant ethnicity (Asian–non-Asian)	− < 0.01	0.02	− 0.04, 0.04	< 0.1	0.978
Mask * stimuli ethnicity	− 0.02	0.03	− 0.08, − 0.05	− 0.6	0.586
Mask * participant ethnicity	− < 0.01	0.02	− 0.03, 0.03	< 0.1	0.963
Stimuli ethnicity * participant ethnicity	0.01	0.02	− 0.02, 0.04	0.5	0.642
Mask * stimuli ethnicity * participant ethnicity	− 0.02		− 0.08, 0.03	− 0.8	0.424

Random effects	Variance	SD
Participant (Intercept)	< 0.01	< 0.01
Participant| mask condition	< 0.01	0.02
Participant| stimuli ethnicity	< 0.01	< 0.01
Participant| participant ethnicity	0.04	0.21
Actor (Intercept)	< 0.01	0.04
Actor| mask condition	< 0.01	0.07
Actor| stimuli ethnicity	0.01	0.12
Actor| participant ethnicity	< 0.01	0.01

## Appendix 6: Fixed and random effects table for the black ethnicity linear mixed effects model

**Table:**

Fixed effects	Estimate	Standard error	95% CI	*t*	*p*
Mask condition (unmasked–masked)	0.23	0.02	0.20, 0.26	13.6	< 0.001
Stimuli ethnicity (black–non-black)	− 0.05	0.03	− 0.10, 0.01	− 1.6	0.134
Participant ethnicity (black–non-black)	− 0.03	0.03	− 0.09, 0.03	− 1.0	0.337
Mask * stimuli ethnicity	0.02	0.03	− 0.04, 0.09	0.7	0.472
Mask * participant ethnicity	− 0.03	0.02	-0.07, 0.01	− 1.4	0.157
Stimuli ethnicity * participant ethnicity	− 0.1	0.02	− 0.04, 0.03	− 0.3	0.789
Mask * stimuli ethnicity * participant ethnicity	− 0.1	0.04	− 0.07, 0.06	− 0.1	0.424

Random effects	Variance	SD
Participant (Intercept)	0.01	0.11
Participant| mask condition	< 0.01	0.03
Participant| stimuli ethnicity	< 0.01	< 0.01
Participant| participant ethnicity	< 0.01	0.02
Actor (Intercept)	< 0.01	< 0.01
Actor| mask condition	< 0.01	0.06
Actor| stimuli ethnicity	0.02	0.13
Actor| participant ethnicity	< 0.01	< 0.01

## Appendix 7: Fixed and random effects table for the white ethnicity linear mixed effects model

**Table:**

Fixed effects	Estimate	Standard error	95% CI	*t*	*p*
Mask condition (unmasked–masked)	0.23	0.02	0.20, 0.26	14.5	< 0.001
Stimuli ethnicity (White–non-White)	0.02	0.03	− 0.03, 0.07	0.8	0.431
Participant ethnicity (White–non-White)	0.01	0.02	− 0.04, 0.06	0.4	0.724
Mask * stimuli ethnicity	− 0.02	0.03	− 0.08, 0.04	− 0.6	0.573
Mask * participant ethnicity	− < 0.1	0.02	− 0.03, 0.03	< 0.1	0.964
Stimuli ethnicity * participant ethnicity	− 0.01	0.01	− 0.03, 0.02	− 0.3	0.755
Mask * stimuli ethnicity * participant ethnicity	− 0.02	0.03	− 0.08, 0.03	− 0.8	0.396

Random effects	Variance	SD
Participant (Intercept)	0.01	0.11
Participant| mask condition	< 0.01	0.04
Participant| stimuli ethnicity	< 0.01	< 0.01
Participant| participant ethnicity	< 0.01	0.02
Actor (Intercept)	< 0.01	0.04
Actor| mask condition	< 0.01	0.06
Actor| stimuli ethnicity	0.01	0.10
Actor| participant ethnicity	< 0.01	< 0.01

## Abbreviations

PCA: Principal component analysis
RADIATE: Racially diverse affective expression
